# Disturbed Glucose Metabolism and Left Ventricular Geometry in the General Population

**DOI:** 10.3390/jcm10173851

**Published:** 2021-08-27

**Authors:** Volker H. Schmitt, Anna-Maria Billaudelle, Andreas Schulz, Karsten Keller, Omar Hahad, Sven-Oliver Tröbs, Thomas Koeck, Matthias Michal, Alexander K. Schuster, Gerrit Toenges, Karl J. Lackner, Jürgen H. Prochaska, Thomas Münzel, Philipp S. Wild

**Affiliations:** 1 Department of Cardiology, Cardiology I, University Medical Center, Johannes Gutenberg University Mainz, 55131 Mainz, Germany; volker.schmitt@unimedizin-mainz.de (V.H.S.); karsten.keller@unimedizin-mainz.de (K.K.); omar.hahad@unimedizin-mainz.de (O.H.); sven.troebs@uni-mainz.de (S.-O.T.); 2 German Center for Cardiovascular Research (DZHK), Partner Site Rhine-Main, 55131 Mainz, Germany; Thomas.Koeck@unimedizin-mainz.de (T.K.); matthias.michal@unimedizin-mainz.de (M.M.); karl.lackner@unimedizin-mainz.de (K.J.L.); Juergen.Prochaska@unimedizin-mainz.de (J.H.P.); philipp.wild@unimedizin-mainz.de (P.S.W.); 3 Preventive Cardiology and Preventive Medicine, Department of Cardiology, University Medical Center, Johannes Gutenberg University Mainz, 55131 Mainz, Germany; anni_remmert@arcor.de (A.-M.B.); andreas.schulz@unimedizin-mainz.de (A.S.); 4 Center for Thrombosis and Hemostasis (CTH), University Medical Center, Johannes Gutenberg University Mainz, 55131 Mainz, Germany; 5 Medical Clinic VII, Department of Sports Medicine, University Hospital Heidelberg, 69120 Heidelberg, Germany; 6 Department of Psychosomatic Medicine and Psychotherapy, University Medical Center, Johannes Gutenberg University Mainz, 55131 Mainz, Germany; 7 Department of Ophthalmology, University Medical Center, Johannes Gutenberg University Mainz, 55131 Mainz, Germany; alexander.schuster@uni-mainz.de; 8 Institute for Medical Biometrics, Epidemiology and Informatics (IMBEI), University Medical Center, Johannes Gutenberg University Mainz, 55131 Mainz, Germany; gtoenges@uni-mainz.de; 9 Institute of Clinical Chemistry and Laboratory Medicine, University Medical Center, Johannes Gutenberg University Mainz, 55131 Mainz, Germany

**Keywords:** prediabetes, type 2 diabetes mellitus, left ventricular geometry, left ventricular hypertrophy, left ventricular concentric remodeling

## Abstract

Background: This study sought to investigate the prevalence and clinical outcome of left ventricular (LV) geometry in prediabetes and type 2 diabetes mellitus (T2DM) and the impact of glucose metabolism on the incidence of left ventricular hypertrophy (LVH). Methods: 15,010 subjects (35–74 years) of the population-based Gutenberg Health Study were categorized into euglycemia, prediabetes, and T2DM according to clinical and metabolic (HbA1c) information. Clinical outcome was assessed via structured follow-up. Results: The study comprised 12,121 individuals with euglycemia (81.6%), 1415 with prediabetes (9.5%), and 1316 with T2DM (8.9%). Prevalence of LVH increased from euglycemia (10.2%) over prediabetes (17.8%) to T2DM (23.8%). Prediabetes and T2DM were associated with increased LV mass index (prediabetes: β1.3 (95% CI 0.78–1.81), *p* < 0.0001; T2DM: β2.37 (95% CI 1.81; 2.92), *p* < 0.0001) independent of age, sex, and cardiovascular risk factors (CVRF). The frequency of LVH was related to the presence of T2DM (prevalence ratio (PR)T2DM 1.2 (95% CI 1.06–1.35), *p* = 0.0038). T2DM was related to mortality independent of age, sex, and CVRF regardless of LVH (hazard ratio (HR)T2DM-LVH 2.67 (95% CI 1.94–3.66), *p* < 0.0001; HRT2DM-noLVH 1.59 (95% CI 1.29–1.96), *p* < 0.0001), prediabetes was only associated with outcome in individuals with LVH independent of age and sex (HRprediabetes-LVH 1.51 (95% CI 1.01–2.25), *p* = 0.045). Neither T2DM nor prediabetes were predictors of incident LVH after adjustment for clinical covariates. Conclusions: Prediabetes and T2DM promote alterations of cardiac geometry. T2DM and particularly the coprevalence of T2DM with LVH substantially reduce life expectancy. These findings highlight the need for new therapeutic and screening approaches to prevent and detect cardiometabolic diseases at an early stage.

## 1. Introduction

Type 2 diabetes mellitus (T2DM) represents one of the key risk factors for the development of cardiovascular disease (CVD). Individuals with T2DM are at a higher risk for developing coronary heart disease [1], heart failure [2], peripheral artery disease [3], and acute cardiovascular events such as myocardial infarction and stroke [4]. Besides the well-known cardiac affection due to the development of coronary artery disease and cardiac autonomic neuropathy, T2DM also represents a risk factor for cardiac hypertrophy, which is highly prevalent even in asymptomatic diabetic patients and strongly associated with an increased risk for heart failure, stroke, and sudden death [5].

A body of evidence suggests that the activation of the renin-angiotensin system and alterations in calcium homeostasis as well as glycosylation of proteins and lipids are main contributors to myocardial fibrosis and promote myocardial hypertrophy [6,7]. Cardiac hypertrophy and fibrosis represent two main structural impairments of the diabetic heart, which are triggered by cardiomyocyte loss with compensatory hypertrophy of resident cardiomyocytes and inflammatory pathways generating fibrosis [5]. Additionally, microvascular endothelial dysfunction and the formation of nitrogen species as well as reactive oxygen seem to play key roles in this myocardial pathology [5,8]. Myocardial alterations lead to abnormal contractile function and can later on result in heart failure [8].

The disease burden is already present in earlier stages of impaired glucose metabolism, since alterations of the heart muscle were already seen in insulin resistance and prediabetes [9], suggesting that the disease burden might be higher than so far presumed. In the prediabetic state, functional myocardial changes without structural modification were described [10], but also an increase of left ventricular hypertrophy (LVH) and left ventricular mass index (LVMI) was detected [9,11]. Raising evidence suggests insulin resistance as a key factor in diabetic cardiomyopathy development, leading to an impairment of myocardial glucose utilization and several further pathways resulting in cardiac stiffness and diastolic dysfunction [5]. Aggravated glucose intolerance was associated with increased left ventricular mass and cardiac wall thickness in the Framingham Heart Study [12] and an association between insulin resistance and the presence of cardiac remodeling and hypertrophy was shown in cardiac magnetic-resonance imaging studies [5,13,14].

However, up to now only a few studies investigated the associations between different diabetic states and different forms of left ventricular (LV) geometry. In the present study, the prevalence of different forms of LV geometry in euglycemic, prediabetic, and diabetic individuals was investigated in a large population-based European cohort under highly standardized conditions. Furthermore, the impact of prediabetes and T2DM on the incidence of LVH was analyzed. Additionally, the impact of impaired glucose metabolism with and without the presence of LVH on mortality was investigated.

## 2. Materials and Methods

### 2.1. Design of the Gutenberg Health Study

The Gutenberg Health Study (GHS) represents a population-based, prospective, observational cohort study in Mid-Western Germany starting in 2007; 15,010 individuals were investigated within a highly standardized study platform. The local ethics committee and the data protection officer approved the study before initiation (reference no. 837.020.07[5555]) and participating individuals provided a written informed consent before study enrolment. All study procedures were performed according to the recommendations for Good Epidemiology Practice and the principles of the Declaration of Helsinki. The design and rationale of the study was published before [15]. Data acquisition, definition of cardiovascular risk factors (CVRF), and echocardiographic assessment are described in detail in the Appendix A Methods.

### 2.2. Definition of LVH and Its Phenotypes

LV mass was calculated using the formula ‘LVM = 0.8 {1.04 [(LVEDD + PWD + SD)^3^ − LVEDD^3^]} + 0.6 g’ and relative wall thickness (RWT) via ‘RWT = 2 PWD/LVEDD’ according to the recommendation of Lang et al. [16]. LVMI was performed by relating LVM to the body surface. LVH subgroups were defined according to Lang et al. [16]: regular geometry was present if LVMI ≤ 95 gm/m^2^ (women) or ≤115 gm/m^2^ (men) and RWT ≤ 0.42. Concentric remodeling was defined as LVMI ≤ 95 gm/m^2^ (women) respectively ≤115 gm/m^2^ (men) and RWT > 0.42. Concentric hypertrophy was present if LVMI > 95 gm/m^2^ (women) or >115 gm/m^2^ (men) and RWT > 0.42. Eccentric hypertrophy was defined as LVMI > 95 gm/m^2^ (women) or >115 gm/m^2^ (men) and RWT ≤ 0.42.

### 2.3. Definition of Prediabetes and Type 2 Diabetes Mellitus

Euglycemia, prediabetes, and T2DM were defined according to the guidelines of the American Diabetes Association [17]. HbA1c was measured with a standardized high-performance liquid chromatography assay (Bio-Rad Laboratories, Hercules, California, USA). Individuals with HbA1c levels < 5.7% (<39 mmol/mol) were defined as euglycemic subjects and prediabetes was present if HbA1c accounted between 5.7% and 6.4% (39–47 mmol/mol). T2DM was defined as presence of HbA1c ≥6.5% (≥48 mmol/mol), preexisting T2DM diagnosis by a physician, or intake of antidiabetic drugs. Individuals with diabetes mellitus other than T2DM (e.g., type 1 diabetes mellitus, gestational diabetes mellitus, and diabetes mellitus following pancreatitis) and euglycemic subjects with normal HbA1c levels but fasting glucose >125 mg/dL were excluded from the present study.

### 2.4. Prospective Analyses on Incident LVH and Mortality Assessment

Cumulative 5-years LVH incidences were analyzed based on the subsample being LVH-free at baseline. All individuals of the study were precisely followed-up including death. Physicians’ records were included if available. Mortality was verified by the mortality register of Rhineland-Palatinate, Germany.

### 2.5. Statistical Analysis

The relationship of cardiac geometry (i.e., LVMI and RWT) was analyzed in multivariate linear regression models adjusted for age, sex, and CVRF and in robust Poisson regression models. Incidence plots for all-cause mortality and cardiovascular mortality according to T2DM and prediabetes with and without LVH were generated. The impact of LVH on mortality was examined in Cox regression models with outcome as the dependent variable adjusted for age, sex, and CVRF stratified for the presence of LVH in individuals with prediabetes and T2DM. Finally, the association of T2DM and prediabetes with different cardiac geometries was analyzed by multivariate regression analyses adjusted for age, sex, systolic and diastolic blood pressure, BMI, continuous measures of dyslipidemia, FH of MI/stroke, and current smoking. Further detail on statistical analysis is provided in the Appendix A Methods.

## 3. Results

### 3.1. Study Cohort and Prevalence of LVH Stratified on Diabetic State

**Figure 1:**
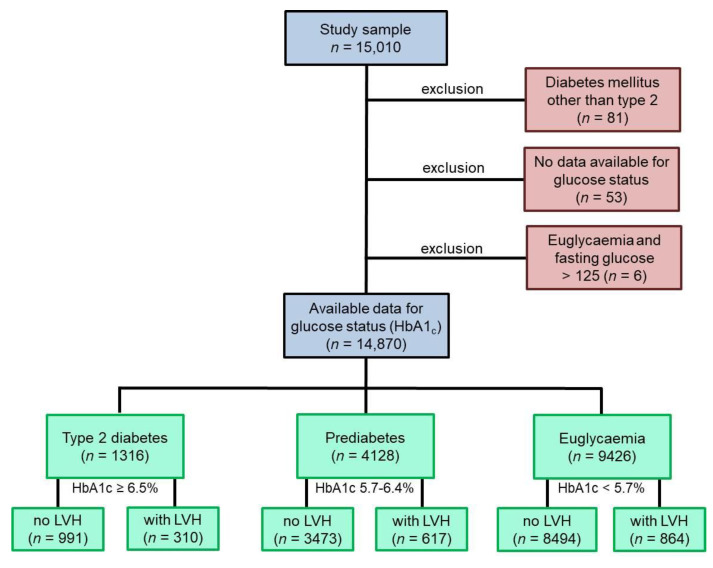
Study flow chart. Blue boxes represent the GHS study cohort, of which the individuals included into the present study were chosen. Red boxes show excluded individuals. Green boxes represent the subgroups of the final study sample. A total of 14,870 subjects were included. Individuals with a type of diabetes mellitus other than T2DM were excluded, also if data for glucose states were not available and in case of normal HbA1c but fasting Glucose >125 mg/dL. No LVH was present if LVMI ≤ 95 gm/m^2^ (women) or ≤115 gm/m^2^ (men) and RWT ≤ 0.42; all other states were defined as present LVH. GHS: Gutenberg Health Study. LVMI: left ventricular mass index. LVH: left ventricular hypertrophy. RWT: relative wall thickness. T2DM: type 2 diabetes mellitus.

From the GHS cohort comprising 15,010 participants, 14,870 individuals were included in the present study (Figure 1); 140 subjects were excluded due to diabetes other than type T2DM, unavailable glucose state or euglycemia, and fasting glucose >125 mg/dL. In 9426 individuals, euglycemic status was present, prediabetes was detected in 4128 subjects, and T2DM was observed in 1316 participants.

Data regarding LVH was present for 9358 individuals in the euglycemia group, accounting 8494 subjects without and 864 individuals with LVH (9.2%) at baseline. Of the 4090 individuals with prediabetes and available LVH data, 3473 subjects were without and 617 participants (15.1%) were with LVH. In the T2DM group, the LVH data of 1301 individuals were available and 991 participants were without whereas 310 subjects were with LVH (23.8%).

### 3.2. Diabetic State and Prevalence of Cardiovascular Risk Factors, Comorbidities and Different Forms of Left Ventricular Geometry

According to the diabetic state and presence or absence of LVH, traditional CVRF, cardiovascular comorbidities, and parameters of cardiac structure are given in Table 1. Compared to individuals without LVH, subjects with LVH were older and had a higher prevalence of CVRF as well as cardiovascular comorbidities in all diabetic states. LVMI was higher in subjects with prediabetes and T2DM compared to individuals with euglycemia, and RWT was highest in individuals with T2DM.

### 3.3. The Influence of the Diabetic Phenotype on Left Ventricular Geometry

With the aim to assess possible associations between the diabetic state and LV geometry, multiple linear regression models were performed (Table 2A). For prediabetes and T2DM, a strong association was present to LVMI after adjustment for sex and age, which persisted after additional adjustment for traditional CVRF. An association between RWT and prediabetes as well as T2DM was present after adjustment for sex and age. After additional adjustment for traditional CVRF, the association persisted in prediabetes and was attenuated in T2DM.

The influence of prediabetes and T2DM on LV geometry was investigated using robust Poisson regression models. Prediabetes was associated with an increased risk for LVH compared to euglycaemic subjects after adjustment for sex and age (PR 1.22 (95% CI 1.10–1.34), *p* = 0.0001) and additional adjustment for CVRF (except for T2DM) (PR 1.12 (95% CI 1.01–1.23), *p* = 0.026), but after further adjustment for inflammation, E/E’ and EF the association was attenuated. In contrast, risk for LVH was increased by 22% in individuals with T2DM independent of age, sex, CVRF (except for T2DM), inflammation, E/E’, and EF (PR 1.22 (95% CI 1.07–1.39), *p* = 0.0029). Individuals with T2DM revealed a higher risk for eccentric hypertrophy of 41%, whereas prediabetes was no independent risk factor after adjustment for of age, sex, CVRF (except for T2DM), inflammation, E/E’, and EF (Table 2B).

### 3.4. Influence of Left Ventricular Hypertrophy on Mortality in Different Diabetic States

**Figure 2:**
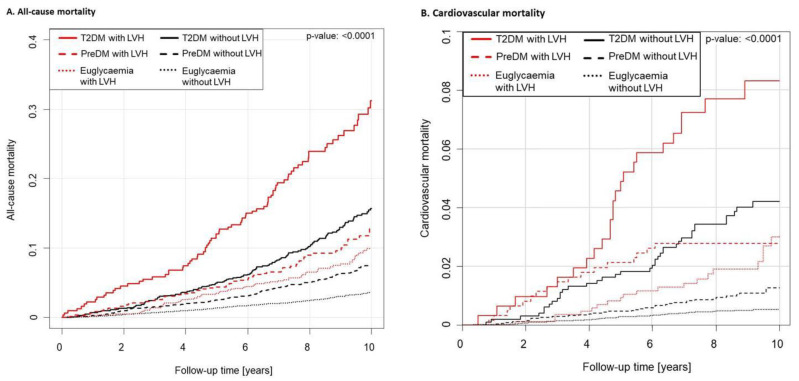
Mortality by diabetic state and presence of LVH. Compared all-cause mortality (**A**) and cardiovascular mortality (**B**) regarding diabetic state in individuals with and without left ventricular hypertrophy (LVH). Mortality in individuals with prediabetes and LVH was as high as in diabetes without LVH. A total of 808 deaths were registered within a median observational follow-up time of 9.04 years (IQR 7.7–10.0 years). In 12,958 individuals without LVH, 596 deaths including 117 fatal cardiovascular events were recorded, and of 1791 individuals with LVH 205 deaths were notified (60 events of cardiovascular reasons). The *p*-value of the log-rank test is given in the figure.

Using Cox regression models, the influence of LVH in individuals with prediabetes and T2DM on mortality was assessed. During a median observational follow-up time of 9.04 years (IQR 7.7–10.0 years), a total of 808 deaths were recorded. In 1791 individuals with LVH survival status was present and 205 deaths were notified, of which 60 events were of cardiovascular reasons. Mortality data of 12,958 subjects without LVH was available (596 deaths including 117 fatal cardiovascular events). Mortality in individuals with prediabetes and T2DM was compared in presence and absence of LVH (Table 3). After adjustment for age, sex, traditional CVRF, and comorbidities, no independent association between prediabetes and death of all-cause was present in individuals with or without LVH. In people with T2DM without the presence of LVH, mortality rate was 63% elevated in comparison with euglycemic individuals after adjustment for age, sex, traditional CVRF, and comorbidities. All-cause mortality in subjects with T2DM and additional LVH was even increased by a factor of 2.2. Cardiovascular mortality was not elevated in individuals with prediabetes regardless of the presence of LVH after adjustment for age, sex, traditional CVRF, and comorbidities. T2DM with and without LVH was associated with a doubled cardiovascular mortality rate compared with the euglycemic state, whereas the rate was lower in presence compared with absence of LVH. The cumulative incidence of all-cause and cardiovascular mortality is illustrated in Figure 2.

### 3.5. Influence of Diabetic State on the Incidence of Left Ventricular Hypertrophy

**Figure 3:**
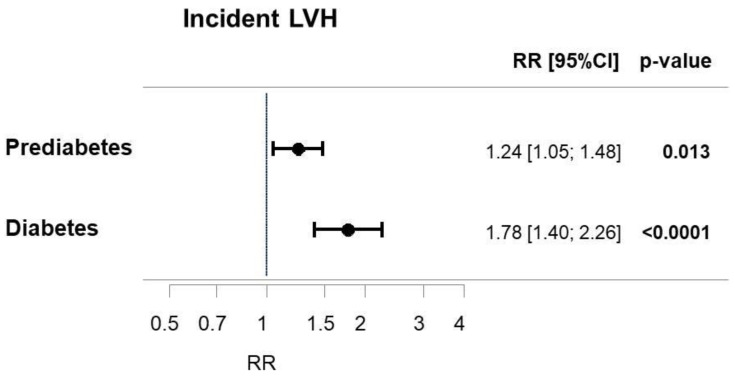
Incidence of LVH in prediabetes and type 2 diabetes mellitus. Illustration of a multiple Poisson regression analyses with incidence of LVH as a dependent variable and prediabetes as well as T2DM as independent variables adjusted for age and sex. CI: confidence interval. LVH: left ventricular hypertrophy. RR: relative risk ratio. T2DM: type 2 diabetes mellitus.

In order to investigate whether the presence of prediabetes or T2DM have an impact on the cumulative 5-years incidence of LVH, prospective analyses using Poisson regression models were performed. Individuals with prediabetes were associated with a 24% elevated 5-years cumulative incidence of LVH, whereas the risk was increased by 78% in individuals with T2DM after adjustment for age and sex (Figure 3). However, after adjustment for age, sex, and CVRF both prediabetes and T2DM revealed no independently associated relative risk for cumulative 5-years incidence of LVH as well as concentric and eccentric hypertrophy (Table 4).

## 4. Discussion

In the present study, the impact of diabetic status on cardiac structure was investigated. In the present findings, the prevalence of LV geometry and its alterations were influenced by prediabetes and in particular by T2DM. Both were associated with larger LVMI and a higher prevalence of eccentric hypertrophy, whereas T2DM was accompanied by higher LVMI and a more pronounced increase regarding prevalence of eccentric hypertrophy than prediabetes. Interestingly, only T2DM had an impact on RWT. It is of high importance that the presence of LVH was associated with a substantial increased long-term all-cause mortality compared to people without LVH in the T2DM population, whereas the risk for cardiovascular mortality was similar in the two groups. In contrast, prediabetes was not responsible for a relevant raise in all-cause as well as cardiovascular mortality, regardless of the presence of LVH. Notably, prediabetes and T2DM had no influence on the incidence of LVH.

Although the prevalence of abnormal LV geometry in people with impaired glucose metabolism was previously described in the literature, beyond that, the present study reported prevalences of all subtypes of altered LV geometry in euglycemia, prediabetes, and T2DM. By this, an elevated prevalence was detected for concentric remodeling and hypertrophy as well as eccentric hypertrophy in both prediabetes and T2DM compared to euglycemia, with concentric remodeling showing the highest prevalence in all groups followed by concentric hypertrophy and eccentric hypertrophy. T2DM revealed the highest prevalence of all forms of abnormal LV geometry. An elevation of concentric remodeling in T2DM compared to euglycemic individuals was also found in a small study including 19 people with T2DM and 19 individuals in the control group [18]. In a multi-ethnic sample, people with T2DM were shown to bare a 1.5-fold risk for LVH, and furthermore an interaction to central obesity was postulated by the authors of this study [19]. In the VALIANT study containing 153 diabetics and 451 non-diabetics, individuals with T2DM revealed an elevated prevalence of eccentric hypertrophy and concentric remodeling compared to euglycemia with almost equal prevalences of both forms of LV geometry (24% and 23%) [20]. Other studies revealed an elevated prevalence of concentric hypertrophy in individuals with T2DM without further analysis of the different forms of LVH [21,22,23].

The present study revealed a relationship between elevated LVMI in prediabetes and T2DM, whereas only T2DM correlated to an increased RWT. An elevation of LV mass in prediabetes and T2DM was also seen in the Strong Heart Study [24,25,26] and an investigation within the Framingham Heart Study cohort revealed an increase of LV mass by 3.0 g for every 1% higher glycated hemoglobin [9]. Interestingly, the MESA study revealed no ethnical differences in the presence of an association between diabetes and LVM, except for Chinese individuals [27]. In the context of RWT, data from the Strong Heart Study [26] and the HyperGEN study revealed an elevated RWT in individuals with T2DM [23] according to the present results. Contrary to the present findings indicating that only T2DM was independently associated with an elevated RWT, this parameter was shown to be associated with both T2DM and prediabetes in the ARIC study with a higher wall thickness in individuals with T2DM [9].

Pathophysiologically, the association between impaired glucose metabolism and altered LV geometry might be in part explained by the hyperglycemia-induced alteration of cardiac metabolism. Elevated glucose levels lead to the formation of advanced glycation end products by non-enzymatic glycosylation of proteins and lipids, which can cause an interference of the myocardial function as well as cross-linking to other proteins like collagen. These molecules stimulate the production of profibrotic factors like Transforming Growth Factor-Beta [7]. Besides advanced glycation end products, hyperglycemia induces an increase in cellular lipid deposits and cardiac hypertrophy caused by increased levels of Insulin-like growth factor [28]. These mechanisms were already detectable in the prediabetic state, which might explain the elevation of LVM and LVMI [7,8,29], as was also shown in an animal model [30]. Additionally, RWT may represent an early parameter of myocardial impairment caused by an increase of protein glycosylation and growth stimulation as a result of elevated blood insulin concentration [24]. Since arterial hypertension represents a common comorbidity of T2DM, it has been postulated that arterial hypertension might be a key factor for the development of LVH in T2DM patients [22]. Data from the HyperGen study revealed a 30% higher LVH prevalence in people with arterial hypertension and T2DM compared to T2DM without arterial hypertension. Nevertheless, an effect on the increase of LVM was also seen independently of arterial hypertension [23]. In the present study, a relevant increase of LVM and RWT in individuals with T2DM was present independently from arterial hypertension, which supports the results from the Strong Heart Study [31]. Of interest, the risk of T2DM-associated complications was accompanied by rising systolic blood pressure [32] and was reduced by consequent anti-hypertensive therapy [33]. Besides hypertension, obesity was also shown to have a high impact on altered cardiac geometry with similar prevalence compared to diabetes [34]. In this context, an association between an increased body fat percentage and elevated LVMI as well as RWT was demonstrated [22] and already obese children revealed an increase of LVMI and had a higher risk regarding concentric hypertrophy [35]. Possible explanations might be the hormone production of adipose tissue which leads to increased levels of angiotensin II and inflammatory cytokines, resulting in cardiac fibrosis and hypertrophy [36,37].

The present study represents the first investigation of the influence of prediabetes and T2DM on left ventricular geometry in a large population-based European cohort of >15,000 individuals including a prospective clinical assessment in a 5-years follow-up. In line with prior findings [38,39,40], prediabetes and T2DM were associated with elevated left ventricular mass. In the present study T2DM, but not prediabetes, was an independent risk factor for developing LVH, challenging future research to clarify the question of a possible impact of prediabetes on cardiac geometry rather than coexisting entities due to an increased risk profile of prediabetic patients. In line, in the present study on a large population-based cohort, prediabetes was not associated to all-cause and cardiovascular mortality in both pre- or absence of LVH, emphasizing the possible sole coexistence of both entities and the need to further assess the role of prediabetes in cardiac geometry. Furthermore, to our knowledge, this is the first large population-based study investigating the incidence of LVH in prediabetes and T2DM. The present results revealed both entities as no independent risk factors for cumulative 5-years incidence of LVH, concentric hypertrophy, as well as eccentric hypertrophy. This suggests, despite the general undisputed severe cardiovascular burden of diabetic disorders, other risk factors like obesity, arterial hypertension, and female sex [41] to have a higher impact on cardiac geometry than glucose metabolism.

Hence, the development of LVH in diabetic people seems to be crucially influenced by the coexistence and quality of therapy of the accompanying risk profile. It has to be emphasized that mortality was crucially increased in the present study in line with prior data in individuals with coexisting LVH and T2DM. These findings highlight the outstanding importance of preventive strategies and early detection of prediabetes, T2DM, and LVH, appropriate management and treatment, as well as continuous monitoring of chronic cardiovascular and metabolic diseases. Early diagnosis and therapy as well as continuous monitoring with optimized medical adjustment have a vast impact on future morbidity and mortality [42].

### Strengths and Limitations

The strength of the present study lies in the large population (>15,000 individuals) with highly standardized clinical and metabolic phenotyping. This involves detailed information for the different forms of LV geometry, which were particularly considered and compared in relation to the presence of prediabetes and T2DM. A study limitation is represented by the lack of other metabolic blood evaluation than HbA1c such as insulin resistance, which has been proven to be linked to cardiac remodeling. Since this study was performed in Germany, extrapolation of the findings to other ethnicities or countries has to be done with caution, as well as to cohorts with varying age ranges. Further studies will be necessary to further investigate the mechanisms of hyperglycemia on the heart muscle in order to improve understanding of diabetic cardiomyopathy and to find pathways which can be influenced by intervention. However, studies like the present investigation are crucial to enhance the understanding of clinical affection and consequences of these disease patterns.

## 5. Conclusions

The present study represents a population-representative investigation on the association between altered glucose metabolism and cardiac geometry. Both are influenced by a complex interplay of various modifications which further may affect each other, which is nowadays not fully understood. T2DM and LVH are associated with an elevated risk for morbidity and death, whereas a combination of both is accompanied by the lowest survival rate. These findings outlined the necessity for further investigations regarding underlying pathomechanisms in order to identify new therapy concepts and emphasize the substantial role of preventive medicine aiming to prohibit and early detect T2DM and other important cardiometabolic disorders.

## Figures and Tables

**Table 1:** Diabetic status and the prevalence of cardiovascular risk factors and comorbidities in people with and without LVH (*N* = 14,852).

	Euglycemia (*N* = 9426)		Prediabetes (*N* = 4128)		T2DM (*N* = 1316)	
	no LVH	LVH	no LVH	LVH	no LVH	LVH
Age (years), mean (SD)	51.4 (10.6)	57.8 (10.9)	58.7 (9.8)	62.8 (8.4)	62.4 (8.5)	64.9 (7.5)
Female sex, % (*n*)	50.7 (4310)	50.8 (439)	49.3 (1713)	54.3 (335)	35.9 (356)	45.8 (142)
Traditional cardiovascular risk factors						
Active smoking, % (*n*)	19.2 (1629)	17.7 (152)	22.0 (761)	16.9 (104)	15.8 (156)	16.9 (52)
Arterial Hypertension, % (*n*)	38.9 (3306)	63.4 (548)	56.7 (1968)	75.0 (463)	77.9 (772)	87.4 (271)
Dyslipidemia, % (*n*)	34.8 (2958)	43.8 (378)	52.8 (1831)	59.9 (369)	75.3 (745)	79.6 (246)
FH of MI/stroke, % (*n*)	20.3 (1724)	24.3 (210)	23.2 (805)	26.4 (163)	27.1 (269)	28.4 (88)
Obesity, % (*n*)	17.7 (1500)	27.9 (241)	28.9 (1004)	38.7 (239)	52.5 (520)	63.9 (198)
Comorbidities						
Atrial fibrillation, % (*n*)	1.6 (133)	5.4 (47)	2.7 (95)	8.4 (52)	4.6 (46)	9.7 (30)
Chronic kidney disease, % (*n*)	0.9 (73)	1.7 (15)	1.1 (37)	0.6 (4)	1.7 (17)	2.3 (7)
Congestive heart failure, % (*n*)	0.6 (49)	3.1 (27)	1.4 (48)	4.2 (26)	1.8 (18)	8.1 (25)
Coronary artery disease, % (*n*)	1.9 (158)	6.0 (52)	5.1 (176)	11.3 (70)	11.2 (111)	20.0 (62)
Myocardial infarction, % (*n*)	1.2 (99)	4.4 (38)	3.4 (117)	8.6 (53)	7.4 (73)	16.1 (50)
Peripheral artery disease, % (*n*)	2.0 (173)	3.2 (28)	4.2 (147)	5.7 (35)	7.5 (74)	10.6 (33)
Stroke, % (*n*)	1.1 (93)	2.1 (18)	2.3 (80)	2.4 (15)	4.4 (44)	6.5 (20)
Venous thromboembolism, % (*n*)	2.9 (248)	4.6 (39)	5.1 (176)	7.2 (44)	5.6 (55)	6.8 (21)
Echocardiographic measurements						
Left ventricular mass index (g/m^2.7^), median (Q1/Q3)	34.1 (29.0/39.3)	52.4 (48.1/57.9)	36.6 (31.4/41.9)	54.5 (50.4/60.8)	39.8 (33.7/45.6)	59.3 (54.0/66.8)
Relative wall thickness, median (Q1/Q3)	0.38 (0.34/0.44)	0.43 (0.38/0.50)	0.39 (0.34/0.45)	0.43 (0.38/0.49)	0.42 (0.37/0.48)	0.44 (0.38/0.50)

Discrete variables are expressed as relative and absolute frequencies; continuous variables are provided according to distribution as mean with standard deviation or median with interquartile range. LVH, left ventricular hypertrophy. FH of MI/stroke, family history of myocardial infarction or stroke. SD, standard deviation. T2DM, type 2 diabetes mellitus.

**Table 2:** The influence of diabetic state on relative wall thickness, left ventricular mass index, and phenotypes of left ventricular geometry.

A. Diabetic State and Continuous Traits of Left Ventricular Mass								
	Relative Wall Thickness				Left Ventricular Mass Index (g/m^2.7^)			
	Model 1: Age, Sex		Model 2: Add. Traditional CVRF		Model 1: Age, Sex		Model 2: Add. Traditional CVRF	
	β (95% CI)	*p*-Value	β (95% CI)	*p*-Value	β (95% CI)	*p*-Value	β (95% CI)	*p*-Value
Prediabetes vs. euglycemia	−0.0072 (−0.010; −0.004)	<0.0001	−0.0111 (−0.014; −0.008)	<0.0001	1.81 (1.44; 2.18)	<0.0001	0.784 (0.43; 1.14)	<0.0001
Type 2 diabetes vs. euglycemia	0.0104 (0.0055; 0.0152)	<0.0001	−0.000008 (−0.005; 0.005)	1.0	5.78 (5.19; 6.36)	<0.0001	2.48 (1.91; 3.05)	<0.0001
**B. Diabetic State and Phenotypes of LV Geometry**								
	**LV Hypertrophy**		**Concentric Remodeling**		**Concentric Hypertrophy**		**Eccentric Hypertrophy**	
	**PR (95% CI)**	***p*-Value**	**PR (95% CI)**	***p*-Value**	**PR (95% CI)**	***p*-Value**	**PR (95% CI)**	***p*-Value**
Prediabetes vs. euglycemia	1.093 (0.988; 1.208)	0.084	0.829 (0.785; 0.874)	<0.0001	0.918 (0.805; 1.047)	0.2	1.103 (0.942; 1.290)	0.22
Type 2 diabetes vs. euglycemia	1.219 (1.070; 1.388)	0.0029	0.957 (0.888; 1.031)	0.25	1.065 (0.898; 1.264)	0.47	1.410 (1.147, 1.733)	0.0011

A. Multiple linear regression models for investigation of the association between diabetic state (independent variable) on relative wall thickness and left ventricular mass index as dependent variables. Model 1 adjusted for age and sex. Model 2 adjusted for age, sex, hypertension, dyslipidemia, obesity, smoking, FH of MI/stroke. B. Robust Poisson regression models for assessment of the influence of diabetic state on phenotypes of LV hypertrophy adjusted for age, sex, hypertension, dyslipidemia, obesity, smoking, FH of MI/Stroke, log(CRP (mg/L)), E/E’, and EF. CI, confidence interval. CRP, C-reactive protein. CVRF, cardiovascular risk factors. FH of MI/stroke, family history of myocardial infarction or stroke. LV, left ventricle. PR, prevalence ratio. vs., versus.

**Table 3:** Influence of left ventricular hypertrophy on mortality in prediabetes and type 2 diabetes mellitus.

	Model 1: Age, Sex		Model 2: Add. Traditional CVRF		Model 3: Add. Comorbidities	
	Hazard Ratio (95% CI)	*p*-Value	Hazard Ratio (95% CI)	*p*-Value	Hazard Ratio (95% CI)	*p*-Value
All-cause mortality in individuals without LVH						
Prediabetes	1.25 (1.04; 1.51)	0.017	1.15 (0.96; 1.39)	0.13	1.13 (0.94; 1.37)	0.19
Type 2 diabetes	2.00 (1.60; 2.48)	<0.0001	1.72 (1.37; 2.16)	<0.0001	1.63 (1.29; 2.06)	<0.0001
All-cause mortality in individuals with LVH						
Prediabetes	1.03 (0.73; 1.47)	0.86	1.02 (0.72; 1.45)	0.90	0.99 (0.69; 1.42)	0.95
Type 2 diabetes	2.47 (1.78; 3.44)	<0.0001	2.43 (1.71; 3.46)	<0.0001	2.18 (1.52; 3.15)	<0.0001
Cardiovascular mortality in individuals without LVH						
Prediabetes	1.22 (0.78; 1.92)	0.39	1.12 (0.71; 1.76)	0.62	1.07 (0.67; 1.70)	0.78
Type 2 diabetes	3.12 (1.97; 4.89)	<0.0001	2.56 (1.55; 4.25)	0.00026	2.41 (1.44; 4.03)	0.00079
Cardiovascular mortality in individuals with LVH						
Prediabetes	0.98 (0.52; 1.87)	0.95	0.92 (0.48; 1.76)	0.80	0.90 (0.46; 1.79)	0.77
Type 2 diabetes	2.20 (1.21; 3.98)	0.0096	1.95 (1.07; 3.57)	0.029	1.91 (1.04; 3.51)	0.038

Cox regression analyses for investigation of mortality in presence and absence of LVH in prediabetes and type 2 diabetes mellitus. Four calculations were performed and each was adjusted for models 1, 2, and 3. Model 1 adjusted for age and sex. Model 2 adjusted for age, sex, hypertension, dyslipidemia, obesity, smoking, and family history of myocardial infarction or stroke. Model 3 adjusted for age, sex, hypertension, dyslipidemia, obesity, smoking, family history of myocardial infarction or stroke, myocardial infarction, coronary artery disease, stroke, atrial fibrillation, peripheral artery disease, venous thromboembolism, chronic obstructive pulmonary disease, and chronic kidney disease. CI, confidence interval; CVRF, cardiovascular risk factors; LVH, left ventricular hypertrophy.

**Table 4:** Relevance of prediabetes and type 2 diabetes for the incidence of LVH.

	Model 1: Age, Sex		Model 2: Add. Traditional CVRF	
	Relative Risk (95% CI)	*p*-Value	Relative Risk (95% CI)	*p*-Value
Incident left ventricular hypertrophy				
Prediabetes vs. euglycemia	1.24 (1.05; 1.48)	0.013	0.94 (0.79; 1.12)	0.50
Type 2 diabetes vs. euglycemia	1.78 (1.40; 2.26)	<0.0001	0.89 (0.68; 1.17)	0.41
Incident concentric hypertrophy				
Prediabetes vs. euglycemia	0.96 (0.55; 1.70)	0.90	0.61 (0.33; 1.13)	0.11
Type 2 diabetes vs. euglycemia	1.74 (0.67; 4.56)	0.26	0.78 (0.24; 2.58)	0.69
Incident eccentric hypertrophy				
Prediabetes vs. euglycemia	1.27 (1.06; 1.52)	0.0097	0.97 (0.81; 1.17)	0.74
Type 2 diabetes vs. euglycemia	1.89 (1.47; 2.44)	<0.0001	0.95 (0.72; 1.25)	0.71

Multivariable Poisson regression models for prospective analyses regarding the influence of prediabetes and type 2 diabetes mellitus on the cumulative 5-years incidence and its subtypes concentric hypertrophy and eccentric hypertrophy. All LVH incident events in the sample were without any LVH at baseline. Model 1 adjusted for age and sex. Model 2 adjusted for age, sex, hypertension, dyslipidemia, obesity, smoking, and family history of myocardial infarction or stroke. CI, confidence interval; CVRF, cardiovascular risk factors; LVH, left ventricular hypertrophy.

## Appendix A. Methods

### Appendix A.1. Data Acquisition

Participants underwent a standardized assessment including measurements of anthropometry (including body weight and height), gathering traditional cardiovascular risk factors (CVRF) (age, sex, arterial hypertension, diabetes mellitus, dyslipidemia, obesity, smoking, positive family history for myocardial infarction or stroke) and clinical comorbidities (including coronary artery disease, myocardial infarction, stroke or transient ischemic attack, heart failure, atrial fibrillation, peripheral artery disease), standardized echocardiography, medication assessment, and venous blood sampling. Individuals with no prevalent CVD were assessed regarding subclinical CVD according to current guideline recommendations [43].

### Appendix A.2. Definition of Cardiovascular Risk Factors

Traditional cardiovascular risk factors (CVRF) included arterial hypertension, diabetes mellitus, dyslipidemia, obesity, smoking, and positive family history of myocardial infarction or stroke. Arterial hypertension was defined as follows: systolic blood pressure ≥ 140 mmHg or diastolic blood pressure ≥ 90 mmHg or hypertension diagnosed by a physician or medication treatment of hypertension (self-reported). Dyslipidaemia was present if: LDL/HDL-ratio >3.5 or dyslipidaemia diagnosed by a physician or medication treatment of dyslipidaemia (self-reported). Obesity was defined as body mass index ≥ 30. Smoking status was differenced into currently active smoking (smoker) or no currently active smoking (non-smoker). Positive family history of disease was present in the case of at least one reported first degree relative with myocardial infarction or stroke at an age ≤ 60 years (male) or ≤65 years (female).

### Appendix A.3. Echocardiographic Measurement

The echocardiographic examination was performed by trained medical-technical assistants using the echocardiography machine iE33 (Philips, Hamburg, Germany) and the ultrasonic probe S5-1 (Royal Philips Electronics, Amsterdam, the Netherlands). In left lateral position parasternal long and short axis as well as two, three, and four chamber views were performed and analyzed. Measurements of LV end-diastolic diameter (LVEDD), posterior end-diastolic wall thickness (EDPWT), and end-diastolic septum thickness (EDST) were measured. LV ejection fraction was evaluated in four chamber view using the Simpson method, for analysis of the diastolic function E and E‘ were measured via pulsed wave doppler and tissue doppler imaging in four chamber view.

### Appendix A.4. Statistical Analyses

Statistical analyses were performed with the statistical software package R (version 3.5.1, R Foundation for Statistical Computing, Vienna, Austria). Mean and standard deviation were used for continuous variables, for skewed distribution (defined as│skewness│ >1) median and interquartile ranges were conducted. Relative and absolute frequencies were used for categorical variables. Statistical comparison of categorical data was performed by Fisher exact or chi-squared tests whereas for continuous variables Mann-Whitney U test or Student t-test were used. To assess the relation of diabetic state and cardiac structure (LVMI and RWT), linear regression models were performed. The investigation of the relation of diabetic state and LVH phenotypes occurred using Poisson regression models with robust variance estimates for prevalence ratios (PR). The models were first adjusted for sex and age and in a second step additionally for CVRF except diabetes mellitus. With Kaplan-Meier curves and Cox regression models, survival analyses were performed. The influence of diabetic state on prospective incidence of LVH was calculated using Poisson regression models. *p*-values were interpreted as continuous measure of statistical evidence due to the explorative character of this study. Further, *p*-values < 0.05 were considered as relevant.
